# The Cultural Evolution of Games of Chance: A Historical Investigation of Chinese Gambling

**DOI:** 10.1007/s12110-024-09471-9

**Published:** 2024-05-31

**Authors:** Ze Hong

**Affiliations:** Department of Sociology, University of Macau, E21B Avenida da Universidade, Taipa, 999078 Macau China

**Keywords:** Gambling, Gaming odds, Historical Chinese games of chance, Probability theory, Wagers, Betting

## Abstract

Chance-based gambling has been a recurrent cultural activity throughout history and across many diverse human societies. In this paper, I combine quantitative and qualitative data and present a cultural evolutionary framework to explain why the odds in games of chance in premodern China appeared “designed” to ensure a moderate yet favorable house advantage. This is especially intriguing since extensive research in the history of probability has shown that, prior to the development of probability theory, people had very limited understanding of the nature of random events and were generally disinclined to think mathematically about the frequency of their occurrence. I argue that games of chance in the context of gambling may have culturally evolved into their documented forms via a process of selective imitation and retention, and neither the customers nor the gambling houses understood the probability calculus involved in these games.

Gambling, broadly defined as the act of wagering or risking something of value on an uncertain event in the pursuit of potential gain, has been a widespread human activity across cultures and throughout history (Schwartz, 2006). Due to its significant social and economic impact, gambling has attracted much scholarly interest, with numerous studies devoted to understanding its causes and consequences (Guillou Landreat et al., 2019; Walker & Barnett, 1999).

The reasoning and behavioral biases of gamblers, such as the gambler’s fallacy (the belief that the probability of an event is lowered when that event has recently occurred) (Clotfelter & Cook, 1993), the hot-hand fallacy (the belief that a person who experiences a successful outcome has a greater chance of success in further attempts) (Ayton & Fischer, 2004), and the illusion of control (the tendency for people to overestimate their ability to control events) (Langer, 1975), have been well documented, and most existing sociological studies have focused on the adverse effects of gambling in contemporary societies (Bergh & Kühlhorn, 1994; Walker & Barnett, 1999). In contrast, the design of the games themselves received relatively less scholarly attention. Even in standard cultural histories of gambling, authors typically provide no explicit theory of why gambling games appeared the way they did. Such omission is unfortunate, because the design of these games can reveal important features of human cognition as manifested in cultural products, similar to how gambling behaviors shed light on decision-making biases of gamblers. This paper aims to fill this gap by adopting a cultural evolutionary framework to explain why the odds in games of chance in premodern China exhibit certain “design” features. Specifically, it explores why the odds were almost always set to guarantee a house advantage,^1^ while ensuring the advantage was not overly large so the gamblers were still sufficiently incentivized to participate. This becomes particularly intriguing given that extensive research in the history of probability theory has shown that prior to the development of probability theory (which was itself inspired by games of chance) people had a very limited understanding of the nature of random events and were generally disinclined to think mathematically about the frequency of their occurrence (Hacking, 2006; Hald, 2005; Kidd, 2020).

My paper presents a twofold argument. First, I argue that during the late Qing and early Republic era (eighteenth to twentieth centuries CE) in China, neither the gamblers nor the gambling houses had a probabilistic understanding of the outcomes in games of chance, despite their widespread popularity. In other words, prior to gambling house managers’ awareness and utilization of probability calculus in setting odds (which occurred relatively recently), gambling houses mostly imitated their more successful peers without realizing the statistical guarantee of a house advantage in the odds. As a result, the odds recorded in historical records were the outcome of such a cultural evolutionary process and display certain “design” features. It is important to note that some of the evidence and conclusions presented are speculative by the standards of rigorous historical scholarship, and my aim in this paper is to propose an interesting possibility that could inspire further scholarly investigations rather than making foregone conclusions.

The rest of the paper is structured as follows. The next section presents a concise background on the premodern Chinese understanding of uncertainty, highlighting how events that a modern reader might perceive as random (and therefore require no further explanation) were interpreted rather differently in the past. The following section provides essential descriptions of several gambling games based on chance and offers a comprehensive analysis of the cognitive, social and cultural factors that influence the odds in these games. Finally, I conclude by critically summarizing my arguments and discussing the epistemic differences in contemporary, modern societies.

## Lack of Probabilistic Knowledge in Premodern China

In his two seminal works on the intellectual history of probabilistic thinking in western Europe, *The Emergence of Probability* (1975/2006) and *The Taming of Chance* (1990), Ian Hacking famously proposed that probability as we understand it today only became a coherent concept around the 1650s. Prior to this time period, many aspects of chance phenomena were noted but not systematically addressed (Franklin, 2001). Although similar studies in non-western cultures are less extensive, there are some accounts of probabilistic thinking in premodern China that support Hacking’s implication that the understanding of the stochastic occurrence of uncertain outcomes as quantifiable and calculable was difficult and came rather late in the history of ideas in human societies. Traditionally, Chinese mathematics had focused on algorithms aimed at solving practical problems since *The Nine Chapters on the Mathematical Art* (*Jiuzhang suanshu* 九章算術) in the second century CE (see Kangshen et al., 2000), and probabilistic theories were not introduced to China until the end of the nineteenth century when Thomas Galloway’s article on probability theory in the eighth edition of *Encyclopaedia Britannica* (also published separately as the book *A Treatise on Probability*) was translated into Chinese as a book, *Jueyi Shuxue* (Bréard, 2009). In comparison to other types of scientific knowledge that spread to China during the late Qing/early Republic era, understanding and appreciating probabilistic formalism was particularly challenging due to a lack of knowledge in the intellectual context in which probabilism emerged in western Europe. In fact, serious study of probability and statistics only began in China in the 1930s (Siu & Lih, 2015).^2^

Elvin (2010) explicitly addressed the question of why premodern China did not develop probabilistic thinking, attributing its absence to the Chinese people’s tendency to view uncertain outcomes in terms of personal luck. This mode of thinking may have a deeper cognitive basis: a brief survey of the ethnographic literature reveals that premodern China was by no means the only society in which luck played a significant role in explaining uncertainties in daily life. For example, the Zulu in South Africa attributed highly variable hunting returns to luck, which may be negatively affected by hunters’ taboo violations (Raum, 2019). The Hausa in northern Nigeria similarly believed that economic outcomes are influenced by “luck” (*arziki*), which was deemed as an attribute that individuals possess (Hill, 1972). More generally, in many traditional, preliterate societies people do not readily distinguish among chance, fate, and luck, and they tend to view all significant events as determined by such knowable and influenceable entities as luck, witchcraft, or karma (Berglund, 1976; Henderson, 1969).

It is easy to see how such deterministic thinking^3^ can influence the way people understand games of chance. In the English language, we now use the word “chance” to describe certain types of games (as in “games of chance”) because we view the processes that produce gaming outcomes as intrinsically random (also known as “aleatory”; see Der Kiureghian & Ditlevsen, 2009) and something that humans cannot get better at predicting or influencing. In contrast, in societies where people view uncertain outcomes as knowable and influenceable,^4^ winning or losing in gambling would be considered within the control of gamblers—at least in theory. As a result, gamblers often resort to all kinds of effort to win, sometimes to the point of desperation. Indeed, the psychology of gambling has been extensively studied to understand the cognitive and behavioral biases of gamblers (Cocker & Winstanley, 2015; Griffiths, 1990; Ladouceur et al., 1995).

In anthropology, the close connection between gambling and divination has been proposed since the time of Tylor (1871), and later comparative ethnographic work has provided further empirical support on the structural similarity between divination and chance-based gambling (Roberts et al., 1959).There could also be attempts to directly influence gambling outcomes via supernatural means: among the Malay, for example, the same charms may be used for both love magic and chance-based gambling (Gimlette, 1923). In China, clear evidence suggests that gambling games such as fantan and proto-lotteries were derived from earlier divinatory practices (Paulès, 2010; Price, 1972), and people definitively considered gambling outcomes as nonrandom, as will be discussed in greater detail in subsequent sections.

## Design Without a Designer: Variation and Selective Imitation in the Odds of Pure-Chance Gambling in Premodern China

### Common Chance-Based Gambling with Fixed Odds

Before delving into the details of my full argument, it is necessary to provide brief descriptions of chance-based gambling games in premodern China. The types of games that I will focus on have two defining characteristics. Firstly, they are games of pure chance or games that can, in principle,^5^ be operated as games of pure chance, meaning that they do not involve skills or strategies, and the outcomes of winning or losing only depend on chance in the modern, aleatory sense. Secondly, they are games with fixed odds, which means that the odds of different wagering options (broadly defined) are predetermined and available to the public at the time the wagers are placed. I will nonetheless discuss chance-based gambling with non-fixed odds for comparison purposes.

#### Fantan

Fantan 番攤 was widely popular in Guangzhou during the late Qing and early Republic era in China. It has clear connection with milfoil divination (Smith, 2021) and a mythical origin that traces back to the Han dynasty (Yang, 1990). The basic game mechanics are simple: the croupier places a small pile of tokens (e.g., copper coins or porcelain buttons) under a cover (so it is impossible for gamblers to count the number of tokens) on a large table, and the gamblers need to bet on the remainder of the total number of tokens divided by four. After all gamblers have placed their bets, the croupier slowly removes four tokens at a time using a stick until there are either one, two, three, or four left. The gambler wins if he guessed correctly, and the gambling house charges a 5–10% commission fee on all winnings. Paulès (2010) provides a more thorough description of the rules of the fantan game, but for our purposes the most important aspect of fantan is that the odds are “fair” in the sense that regardless how gamblers place their bet, the expected return is always zero. However, because of the fixed percentage that the house charges on winnings, the house still has an advantage whose magnitude is simply the percentage charged. Similar games of pure chance, such as Ya Bao 押寶, also exist, where the house advantage is likewise implemented through fixed commission fees.^6^

#### “White Pigeon” Lottery

Though lotteries are often viewed as different from “gambling” in many modern societies because of their legal status (often being implemented or at least heavily controlled by the state), they share many essential features as chance-based gambling and historically were often classified as the same type of activity as casino-style gambling games. “White pigeon” lottery 白鴿票 has sometimes been suggested to be the original keno-style lottery, where players select from a pool of available numbers and hope that their chosen numbers will be drawn as the winning numbers via some random process (Bollman, 2018). In its original form, players selected 10 out of 80 characters (taken from the *Thousand Character Classic *千字文), and a professional manager from the lottery company would draw 20 winning characters. Players’ winnings were determined by the number of matches between their selection and the 20 winning characters using a table with fixed odds. Culin (1891) gave a thorough description of the lottery as played in Philadelphia in the late nineteenth century, and Table 1 shows the odds as well as players’ chance of winning and expected returns based on his description.

**Table 1:** “White pigeon” lottery: Chance of winning, odds, and expected return for correcting guessing various numbers of characters according to Culin’s description. The house advantage (after charging the 5% commission fee on winnings) is 1−(0.7996*0.95) = 24.04%. See Bollman (2018) for the mathematical details on how the chances of winning are calculated

Number of winning character catches (out of 10)	Chance of winning	Odds	Expected return
5	5.1428*10^−2^	2/1	0.1029
6	1.1479*10^−2^	20/1	0.2296
7	1.6111*10^−3^	200/1	0.3222
8	1.3542*10^−4^	1000/1	0.1354
9	6.1206*10^−6^	1500/1	0.0092
10	1.1221*10^−7^	3000/1	0.0003
Overall expected return ($1 stake)			0.7996

According to Culin (1891), the selection process is effectively random and occurs in public; in its ancestral form, the 20 winning characters were hand-picked by the manager in private and then placed in sealed containers in public (Guo & Xiao, 1995; Liu & Wang, 1992). Once the managers had picked the winning characters with certain restrictions, the players were basically “guessing” the picks by the manager.

#### “Flower” Lottery

“Flower lottery” 花會 (also known as 會局 or 字花) was another form of lottery that was extremely popular during the late Qing/early Republic era, and its influence went beyond China as Chinese immigrants traveled around the world. In Cuba, it was described as “the cancer of the popular economy” (Caillois, 1957), in Singapore it was “indulged in by all classes . . . to a fearful extent” (Vaughan, 1879), and in Malaysia it was “considered to be the worse [*sic*] kind [amongst all types of gambling]” (Kynnersley, 1885). In flower lottery, the manager from the lottery company would select 1 out of 36 mythical figures (in practice, the total number of possibilities ranges from 34 to 37) as the winning figure, and if the players also picked the winning figure they would receive 30 times their stake as prize money. As in white pigeon lottery, winning figure selection was done by a human rather than a random process.

Human involvement in selecting winning characters/figures in white pigeon lottery and flower lottery means that the selection processes were not, strictly speaking, random. In fact, in flower lotteries the company would often announce a riddle whose answer is presumably the winning figure. However, because the composition of the riddles was such that virtually any figures could be said to be the answer, the game effectively became one of pure chance for the gamblers (Barnett, 1962). The significance of this aspect will be elaborated in later sections.

## Lotteries with Non-fixed Odds

During this time, two other lotteries occurred which were similar to the white pigeon lottery but differ in a few critical aspects. The “mountain lottery” 山票 and the “shop lottery” 鋪票 both required players to pick a certain number of characters from a total of 120 characters, and the winning characters were chosen via a random process in a public setting. Rather than using fixed odds to assign prize money, the lottery companies would allocate a fixed percentage (65–80%) of total wagers as prizes to be distributed among winners (those with the most matched characters). Within the pool of prize money, 60% was allocated as first prize, 25% as the second prize, and 15% as the third prize (Liu & Wang, 1992).

## The Prevalence of Gambling in Premodern China

It is a cliché that the Chinese were historically a gambling people (Paulès, 2010; Riis, 2011). However, much of the documented accounts focused on gambling activities in Canton (now Guangzhou), the only trading port that handled foreign commerce since 1757 under the Single-Port Commerce System (Dong, 2019). The thriving commercial activities undoubtedly led to a boom in gambling, given that numerous authors during the nineteenth and early twentieth centuries noted the Chinese people’s fervor in gambling in this region. Osmond Tiffany, an American merchant, made the following observations during his visit to Canton:

Gambling, I am sorry to say, occupies much of the time that people devote to amusement; there are hundreds of modes of gambling and sums are staked from a few cash up to large sums of money. The boys learn gambling as soon as they can talk, and pursue it through life (Tiffany, 1849).

John Gray, the archdeacon of Hong Kong, made similar remarks:

No amusement is more popular among the Chinese than gambling. The inordinate love of plays is so deeply rooted in the breasts of the people that men and women of all classes of society, and of almost all ages, are gamblers. The gaming houses are numerous everywhere, and are thronged with players (Gray, 1878).

Such descriptions were not limited to foreign accounts. Chinese intellectuals of that time also expressed concern about the excessive gambling habits of the Cantonese people and the social vices it engendered. The scholar Xu Ke in the early twentieth century explicitly described and condemned the gambling habit of the Cantonese:

Cantonese people have a natural tendency towards gambling. They start with betting on surname guessing^7^ and white pigeon lottery, and then move on to fantan and mountain lottery. They can spend the whole day immersed in it, oblivious to the world around them. The lower class is particularly addicted to it (Xu, 2010).

These early accounts often involved (self)stereotyping of the Chinese people and were far from unbiased, objective descriptions. However, the crucial insight for our purposes is that gambling was a significant cultural activity, and there was a large demand for it. While some recent scholars have highlighted the social aspects of gambling, suggesting that it can provide social and emotional benefits to the participants (Saldanha et al., 2018) and that gambling houses (in the physical sense) served as venues for socialization (Li, 2015; Paulès, 2010, 2017), it is crucial to remember that the primary reason for people to gamble was their desire to win. Barnett (1962) aptly summarizes this when describing the gambling behavior of overseas Chinese communities:

Chinese attitudes toward the attainment of wealth enhances compulsions to gamble. This purely economic motive is of sufficient force to make one thing certain: the Chinese plays to win and “for keeps.” Any diversion derived from gambling appears to be secondary. . . . No point is seen in merely playing for “fun.”

Even from a commonsense perspective, losing money is not enjoyable (and most gamblers would likely lose money in the long run), not to mention that the stakes could be as dire as one’s own enslavement or one’s spouse being forced into prostitution (Price, 1972).

## Gamblers and Gambling Houses’ Failure to Understand Outcomes as Random

One might expect that the prevalence of gambling games, many of which rely on chance, would have led to the development of sophisticated theories of probability, as occurred in western Europe in the 1650s. While a thorough investigation of the sociocultural-economic factors that hindered the development of probabilistic theories of uncertainty is beyond the scope of this paper,^8^ it suffices for our purposes to empirically suggest the lack of probabilistic thinking and its sociocultural consequences.

It is well-established that many gamblers do not think probabilistically when they engage in games of chance, and instead attempt to identify and take advantage of patterns or regularities in gambling outcomes when none actually exist (Benhsain et al., 2004). This type of irrational behavior has been studied extensively by economists and psychologists, who have identified various cognitive biases and heuristics that lead people to overestimate their chances of winning (Ayton & Fischer, 2004).

The situation in premodern China appears to have been even more extreme in this regard as patterns and regularities in gambling outcomes were widely believed to exist, despite being difficult to know with certainty. In games such as fantan and white pigeon lotteries, where outcomes are determined purely by chance, gamblers would search for patterns in the sequence of past winning numbers or characters in hopes of predicting future ones. In fantan, these patterns were known as “the ways” 路, upon which gamblers could base their bets (Liu & Wang, 1992; Luo, 1928; Wang, 1932). Similarly, in white pigeon lotteries, the lottery company would print out previous winning characters and display them prominently to incentivize participation based on the belief that there were discernible patterns or trends in the game that could be exploited (Liu & Wang, 1992).

**Fig. 1:**
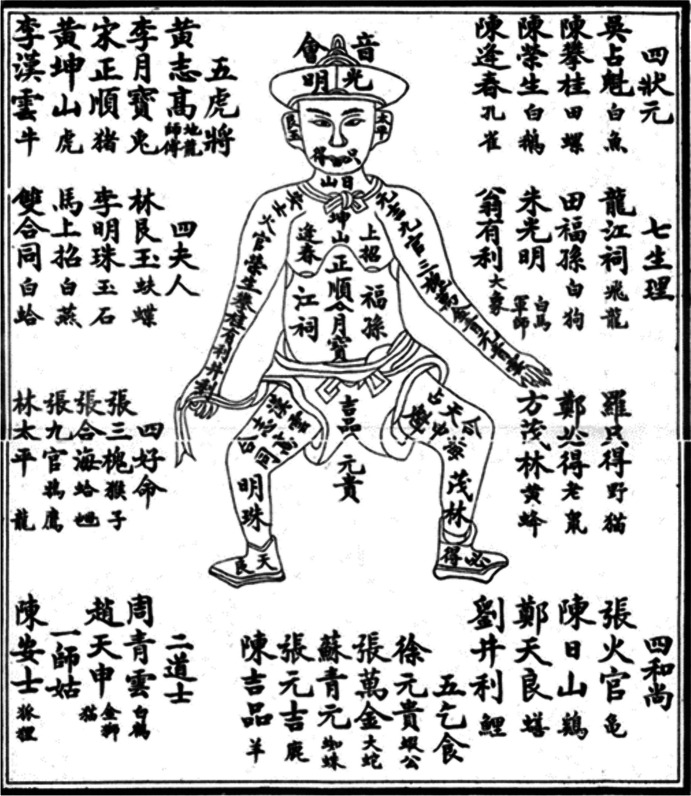
Dream interpretation manuals for flower lottery picks (adapted from Kynnersley, 1885)

An additional factor to consider is that the cognitive life of both commoners and elites in traditional China was heavily influenced by a supernatural worldview, which allowed for events with no apparent physical or mechanistic connection to be perceived as causally associated (Hong & Henrich, 2021).^9^ This type of thinking is sometimes referred to as “magical thinking” (Rosengren & French, 2013) and is common in many traditional societies where supernatural beliefs and practices are widespread. In the context of gambling, this type of magical thinking gave rise to a number of superstitious beliefs and practices among gamblers. For example, fantan players adhered to various taboos in order to ensure good luck and avoid bad luck, such as avoiding uttering certain words that sound like “losing” (Culin, 1891; Paulès, 2010). Similarly, various forms of divination were commonly used to aid gamblers’ lottery picks (Guo & Xiao, 1995). Dreams, in particular, were frequently interpreted as “signs” that foretold the winning figures in flower lotteries, and gamblers would go to desperate lengths to induce dreams, such as sleeping with dead bodies (Liu & Wang, 1992). The popularity of dreams as predictive devices was also manifested in printed pamphlets with pictures that associate each of the lottery figures with different body parts (Fig. 1), the idea being that if one dreams of certain body part then they should bet on the corresponding figure (Kynnersley, 1885). Although these divinatory practices occasionally provided accurate information, they were often ineffective and therefore unreliable. Nevertheless, gamblers would selectively remember and retell their predictive successes while ignoring failures (Zheng & Hu, 1923),^10^ a reporting bias that can contribute to the persistence of ineffective technologies that I discuss extensively elsewhere (Hong, 2022b; Hong & Henrich, 2021).

What about gambling houses? Did the managers have knowledge of probability theory and benefit from the asymmetry of information? In modern casinos, there is little doubt that the odds are carefully calculated by mathematicians (Bollman, 2018), and historical evidence suggests that the designers of keno-style lotteries in western Europe as early as 1758 clearly understood the probability calculus involved (Stigler, 2022). Note that this does not imply that everyone in the society was proficient in probabilistic calculus; rather, it was likely that only a few individuals possessed such expertise (one could argue that culturally transmitted knowledge is never evenly distributed, and individuals are fully aware of this epistemic reality; see Keil et al., 2008). Nonetheless, the existence and systematic transmission of such knowledge was sufficient to influence the setting of odds.

The situation in premodern China, however, was a lot less clear. I propose that, similar to the gamblers, the gambling houses did not consider the probabilistic nature of gambling outcomes, and in particular were not calculating expected returns when setting odds. This argument is based on the following reasons. First, the organizers of gambling houses were influenced by the same supernatural worldview as the gamblers. There is evidence suggesting that even the managers of flower lottery companies adhered to culturally transmitted taboos when selecting winning figures (Ma, 1938) and worshipped the very same deities as gamblers (Fan & Zheng, 2010). More dramatically, Culin (1891) made the remarkable observation that Chinese gambling houses on the East Coast of the United States would deliberately decorate their rooms in white, the color that is typically associated with the dead and therefore would bring bad luck to gamblers:

All colors, save white, are carefully avoided by the owners on the walls and in the decorations of the gambling rooms. White, the color of mourning, the color of the robes thought to be worn by the spirits of the dead, always considered inauspicious, is associated with the idea of losing money, and is believed to bring bad fortune to the patrons, with corresponding gains to the houses. Even the inscriptions to the tutelary spirit are always written on white paper and white candles burned before his shrine (Culin, 1891).

Ironically, a gambling house manager who was knowledgeable about probability would decorate the room in red to attract superstitious gamblers. However, since the house and the gamblers were often in zero-sum situations, the house believed that it could increase its profit by reducing the luck of the players. This suggests that, like the gamblers, gambling establishments also attempted to improve their chances through supernatural means.

Second, gambling houses were known for their attempts to cheat (Guo & Xiao, 1995; Liu & Wang, 1992). Although the extent of cheating may have been exaggerated in more recent accounts for political purposes (e.g., justifying the suppression of gambling), widespread reference to it suggests that it likely occurred to a non-negligible degree. From a rational standpoint, cheating is a bad move for gambling houses as long as they have a probabilistic advantage (which was the case for most gambling houses during this period in China; see the next section). After all, gamblers were never passive victims of house cheating (Paulès, 2010), and the gambling houses’ reputation could be irreparably tainted if cheating was exposed. In a competitive market, such a scandal could even lead to the business’s closure. Elvin (2010) rightly points out that given all the downsides of house cheating, the fact that it still occurred in premodern China indicates that some gambling houses must have felt insecure about potential heavy losses and therefore resorted to last-minute cheating, revealing a lack of understanding of the laws of probability.

We can gather further insights by comparing the selection of winning characters and prize allocation in the four different types of lotteries (Table 2). Note that for the mountain and shop lotteries, whose winning characters were randomly determined in public, a fixed percentage of total ticket sales was allocated as prize money. However, for the white pigeon and flower lotteries, whose winning characters were hand-picked by house managers, fixed-odds payout schemes were used. Was this merely a coincidence? One possibility is that when the total prize money was a fixed percentage of total ticket sales, the house was guaranteed a profit. Consequently, the method of selecting winning characters had no impact on the house’s profit, and they could “afford” to have the selection made randomly. Conversely, when the prize money was determined by fixed odds, the house could theoretically suffer significant losses. Thus, the house felt the need to “outsmart” gamblers by having a person select the winning characters,^11^ indicating that the house did not realize it had a statistical advantage over the gamblers in the long run.

**Table 2:** Methods of winning characters’ selection and prize allocation schemes for four different types of lotteries in late Qing/early Republic China. Information compiled from Guo and Xiao (1995) and Liu and Wang (1992)

	Shop lottery	Mountain lottery	White pigeon lottery	Flower lottery
Winning characters’ selection	Determined randomly in public	Determined randomly in public	Determined by the gambling house manager in private	Determined by the gambling house manager in private
Prize allocation	Percentage of sales	Percentage of sales	Fixed-odds	Fixed-odds

Guo and Xiao (1995) provide a vivid description of the ongoing mental battle between lottery managers and the gamblers as they attempted to outsmart each other:

There is no discernible pattern in the white pigeon lottery. Both the lottery companies and gamblers are trying to guess each other’s mentality, especially in the intense psychological warfare between heavy gamblers and lottery managers. Gamblers buy tickets in the hope of winning, while the lottery managers issue tickets (pick the winning characters) with the aim of making a profit. Therefore, gamblers carefully study the various characters that the managers have picked in the past, looking for patterns . . .  [and] the lottery managers also seek to identify trends based on gamblers’ historical picks and skillfully avoid [their future picks].

Similar descriptions^12^ can be found for flower lotteries^13^ and other lotteries where house managers hand-picked the winning characters.^14^ While attempting to outsmart the house can be intellectually satisfying and enjoyable, many gamblers would likely switch to other gambling houses if they became aware that a house was constantly trying to exploit them. From the house’s perspective, trying to predict or influence the picks of gamblers is also not always the best move; in game theory, random choices can often be optimal in mixed-strategy scenarios (Straffin, 1993). Again, the prevalence of such descriptions, akin to house cheating, suggests that gambling houses likely intentionally sought to avoid players’ picks to a significant degree.

**Fig. 2:**
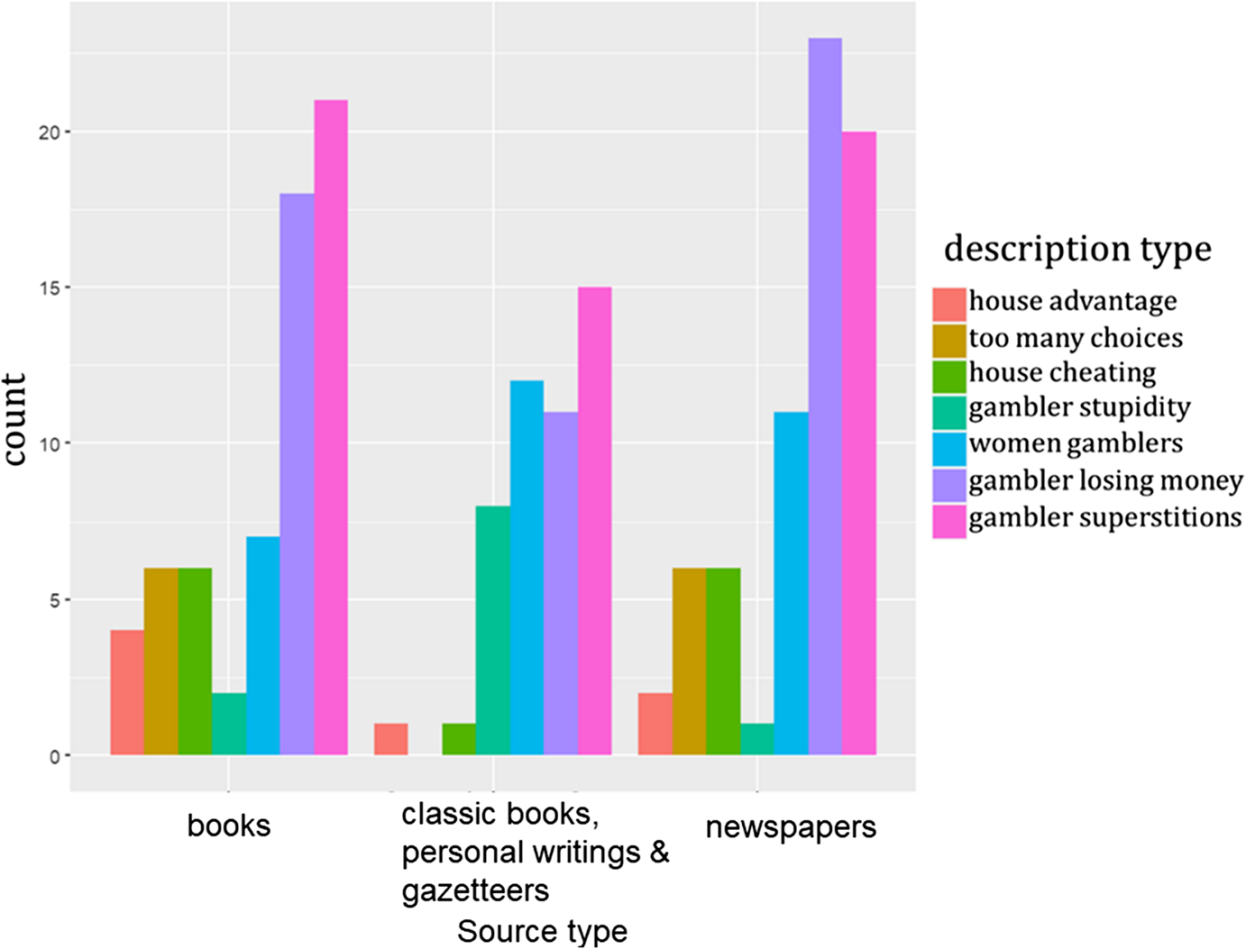
Types of criticisms/negative descriptions of the flower lottery from three different sources. Note that one article may include multiple negative descriptions

Finally, although there is little direct textual evidence on how gambling managers perceive their games, numerous accounts exist of criticisms directed toward gambling from intellectual elites. To gain a better understanding of how critics depicted gambling during this period, I focused on the simplest form of lottery with fixed odds, the flower lottery. I performed keyword searches for “flower lottery” (花會) and “thirty” (三十) in Shanghai Library’s collection of newspapers and books for the late Qing and Republic periods, as well as Erudition’s collection of classic ancient books, local gazetteers, and personal writings. Note that in Chinese, “thirty” (三十) is also the prefix for numbers between thirty and thirty-nine, meaning that a keyword search for “thirty” would also include hits for thirty-one, thirty-two, and so on. Specifically, I examined the types of negative descriptions of this lottery and the extent to which the house advantage was mentioned. Figure 2 shows the breakdown of all descriptions.^15^ Notably, this already overestimates the proportion of references to the house advantage because all flower lottery descriptions here included either the total number of possible picks, odds of winning, or both. Criticisms that did not mention these numeric features (and therefore the house advantage) of the flower lottery were not included in the dataset.

Even in this dataset, which explicitly mentions the numeric features of the games, the majority of gambling critics did not refer to the “house advantage. Instead, their criticism was directed toward other aspects of gambling, such as its negative social effects. Only 7 of 94 articles (less than 8%) made allusions to the probabilistic advantage of the house, and these references were typically vague and/or confused. For example, Bao (1925) made the following comment on house advantage in a very loose sense:

think about it; there’s a total of thirty-six figures and the winning payout odd is only thirty or even twenty-eight. How much the gamblers would lose . . . there are two figures that are never “open” and therefore only thirty-four possibilities, but if we do the calculation the gambler still suffers a loss.

We get no explanation for how such calculation is performed. In fact, this is already the *clearest* exposition of house advantage, relatively speaking. It is unlikely that a rough probabilistic understanding was common enough that the author felt no need to explicitly invoke the math.

During the same time period, numerous descriptions attributed the gambler’s long-term financial ruin to the difficulty of selecting the correct figure among more than thirty possibilities:

If you bet one coin, you can win thirty coins. If you bet one hundred coins, you can win three thousand coins. There are thirty-six possibilities; even if there are a few possibilities that are not “open,” there are still [more than] thirty possibilities. If we think about this, how difficult it is [to pick the winning characters]! Moreover, nine out of ten flower lottery companies cheat (Huang, 1917).

Accounts such as this are by no means rare, where the number of possibilities and odds are explicitly mentioned without any indication of something remotely resembling an expected return. There are also records that directly reveal the critics’ ignorance of the concept of house advantage:

One can win thirty-four times the bet, and the winnings can be collected immediately, which is quite fair. However, trying to win among thirty-four possibilities is an extremely difficult task. Therefore, the flower lottery is purely based on coincidence and should not be trusted (Wang, 1932).

Again, the task is deemed “extremely difficult” due to the large number of possibilities (34) to choose from. Particularly notable in this quote is that the author’s belief that the odds for winning is thirty-four times the bet. Even assuming, for the sake of argument, that the odds were indeed thirty-four times (in reality commission fees on winning were likely charged), in such a case, the lottery’s odds and payout scheme would also be “fair” from a probabilistic perspective, whereas the author only noted the fairness of the immediacy of payouts and concluded that the flower lottery should not be trusted.

## Competition, Variation, and Selective Imitation/Retention of Odds in Games of Chance

As in any other type of business, gambling houses and lottery companies are under pressure to remain profitable, and setting the correct odds is one of the key factors for achieving financial success. However, these establishments face a trade-off when setting the odds. On the one hand they aim to set the odds more favorably to themselves to increase per-customer profits; on the other hand, they seek to offer odds that are favorable to the customers to encourage more participation. Therefore, it is not immediately obvious what the optimal odds should be. This was especially challenging in premodern China since gambling houses did not consider gambling outcomes as probabilistic. Yet, historical records show that the empirical odds of games exhibit remarkable “design features.” Specifically, these odds consistently guaranteed a house advantage (resulting in a negative expected return for gamblers), yet they were not so overwhelming as to deter people from engaging in gambling. In Table 3, I compiled detailed descriptions of gambling games from a few different types of sources (one classic anthology, one modern encyclopedia-style book on traditional Chinese gambling, and two academic articles on Chinese gambling overseas^16^ by an American ethnologist) to give a comprehensive overview of the key features and computed the house advantages. As can be seen, most of the games of chance had positive house advantages between 5 and 30%, except for the game of Pát Chá as described by Culin (1889).^17^ Unfortunately, no further record of this game is available, and it is likely that Culin mis-recorded the rules of this game, or it could be an example of a “mutant” in the cultural evolutionary process that would suffer a selective disadvantage in the long run.

**Table 3:** Brief descriptions of various games of chance in the late Qing/early Republic period

Game	Key features	House Advantage	Time period	Location	Reference
Fantan (番攤)	Fair odds; 7–10% commission fee on winnings	7–10%	Qing dynasty	Guangdong	(Xu, 2010)
Ya Bao (押寶)	Fair odds; 5% commission fee on winnings	5%	Republic era	Northeast China	(Guo & Xiao, 1995)
Flower Lottery (花會)	Winning chance 1/36; odds 30 to 1	16.6%	Qing dynasty	Guangdong	(Xu, 2010)
Flower Lottery (花會)	Winning chance 1/34; odds 30 to 1	11.8%	Qing dynasty	Zhejiang	(Xu, 2010)
Flower Lottery (花會)	Winning chance 1/34; odds 28 to 1	17.6%	Qing dynasty	Shanghai	(Xu, 2010)
Hui Ju (會局)	Winning chance 1/37, odds 30 to 1	18.9%	Republic era	Heilongjiang	(Guo & Xiao, 1995)
Hui Ju (會局)	Winning odds 1/40 with payout 30 to 1	25%	Republic era	unknown	(Guo & Xiao, 1995)
Pat Cha	Fixed-odds dice game	−8.1%	Qing dynasty	USA	(Culin, 1889)
Teetotum	Six-sided spin; winning chance 1/6, odds 4 to 1	33.3%	Qing dynasty	USA	(Culin, 1889)
White Pigeon Ticket/Pak Kop Piu (白鴿票)	Fixed-odds with house advantage; 5% commission fee on winnings	24.0%	Qing dynasty	USA	(Culin, 1891)
Zidan (字膽)	Lottery with Fixed-odds; winning chance 1/80, odds 60 to 1	25%	Republic era	Macau/Guangzhou	(Guo & Xiao, 1995)

**Fig. 3:**
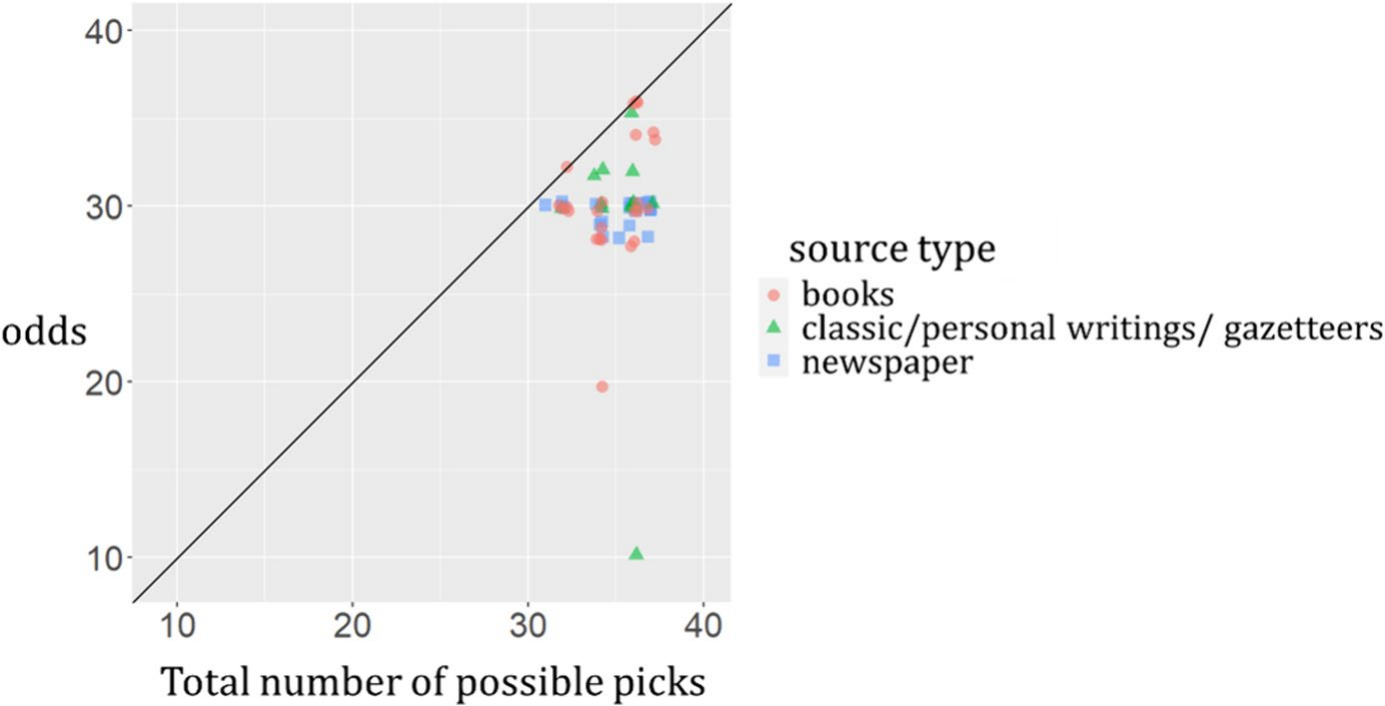
Total number of possible picks vs. odds (the ratio of the possible net profit to the possible net loss) for flower lottery in different sources (*n* = 61). One extreme outlier (odds = 720) was excluded from the analysis

To obtain more fine-grained data on the variation in house advantage in games of chance, I focused on the flower lottery using the same dataset used above to compile Fig. 2. In general, lottery companies had the flexibility to set up their own odds, and the entry barrier for opening a flower lottery shop was particularly low: anyone who claimed to have enough financial ability to distribute the prizes could open a lottery shop (Li, 2015). To examine the extent to which the odds and winning chances of different flower lottery shops differed from each other, I plotted the total number of possible picks against odds (the ratio of the possible net profit to the possible net loss) as documented in various sources (Fig. 3). Figure 3 shows that all data points are either on or below the diagonal line which represents lotteries with fair odds, meaning that the house always had an advantage over gamblers.^18^ More notably, except for two obvious outliers, the house advantages of most of the flower lottery games were within a rather narrow range (0–22%), as can be measured by the distance of the data points to the diagonal line.

Given that most gambling houses did not view games of chance as probabilistic, how could such games exhibit such design features? The extensive literature in cultural evolution provides a plausible explanation. Much theoretical and empirical work has demonstrated that human culture can be understood and investigated in similar ways as genes (Boyd & Richerson, 1985; Henrich, 2016), and cultural products can adaptively evolve in the absence of individuals’ causal understanding (Derex et al., 2019; Harris et al., 2021; Hong & Henrich, 2023). In the context of gambling games of chance, “naive” gambling houses could observe certain existing houses being more successful (e.g., profitable) and selectively copy their game setup, including the odds. Less successful houses were less likely to be copied and could even go bankrupt, which was a real possibility (Barnett, 1962).

Therefore, cultural evolution would produce population-level outcomes very similar to those resulting from individual rational decision-making (where individuals by definition have access to probability theory) in market settings (Nelson, 2007).^19^ Stigler (2022) points out that the design of lotteries should maximize their attractiveness to the bettor, subject to constraints on the need for a profit. This is generally true for games of chance: in a competitive gaming market, standard games with house advantages near or exceeding 40% would be untenable since a rival game operator could easily offer more favorable odds to the players while still making a profit (Bollman, 2018).^20^ How competitive were the gambling markets during the late Qing/early Republic period? Direct evidence of competition is scant, but indirect records suggest that gambling markets were large and likely quite competitive. In addition to the obsessive gambling among the Cantonese as noted by many foreign observers and Chinese intellectuals, numerous references attest to the large number of gambling houses. For instance, the gazetteer of Panyu 番禺 county during the Tongzhi 同治 period (1861–1875) has the following record:

The gambling atmosphere in Guangzhou is very strong. . . . There are more than a hundred fantan houses in the city, and the rural areas are also affected and have almost nowhere without them (Li & Shi, 1871).

Similar situations were observed in Chinese communities abroad as well. Liang Qichao, the famous political activist, remarked on the popularity of gambling during his trip to North America in 1903:

there is not a single household that does not gamble. In the Chinatown area in Vancouver there’s already over twenty gambling houses for fantan and sixteen or seventeen “white pigeon” lottery companies. The situation is similar in other cities as well (Liang, 1967).

We do not have explicit records for gambling houses copying each other’s odds,^21^ possibly because this was such common sense that the writers at the time did not consider it worth mentioning, or because the practice was not openly acknowledged due to the unwillingness to acknowledge a lack of originality in a competitive market. However, in an open market where the odds information was completely public, gambling houses had every incentive to imitate their more successful peers. Indeed, as Nelson (2018) notes, even in modern capitalist economies, evolutionary dynamics could occur when economic actors lack a strong understanding of the context that they are in and of appropriate actions to take.

## Discussion

I have presented two interrelated arguments on games of chance in premodern China; one cognitive and the other cultural evolutionary. The cognitive argument mainly concerns individuals’ perception and understanding of uncertain yet significant outcomes, which aligns with Hacking’s (1990, 2006) thesis that historically people did not have the concept of probability. Although Hacking’s analyses exclusively focus on western Europe, it is plausible that his insights generally apply to traditional societies where uncertain events were predominantly seen as deterministic and knowable (Hong, 2024). In the context of pre-probabilism gambling games, the issue becomes more complex. It is not just that people failed to differentiate between aleatory and epistemic uncertainty (which they most likely did), but more importantly, neither the gambling houses nor the gamblers viewed the outcomes of games of chance (as we would classify them today) as subject to mathematical analysis. This could be due to the lack of technical expertise: it is not surprising that people in premodern China could not compute the expected return of the white pigeon lottery because they lacked the necessary training in combinatorics. However, at a more fundamental level, they did not have the habit of thinking of the winning chances and odds as calculable and comparable ratios, as exemplified in the deceptively simple flower lottery.

This cognitive argument serves as the basis for the cultural evolutionary argument. In premodern (and modern) China, gambling in almost all forms was consistently viewed negatively by authorities, despite occasional legalization for taxation purposes (Li, 2015). Nonetheless, the games survived and thrived all the way into the Republic. From the perspective of economics, gambling houses could be viewed as providing goods and services (gambling opportunities) to customers (gamblers), and the strong demand (people’s gambling desires) contributed to their persistence in Chinese society. The gambling houses, however, faced the challenge of setting odds for games of chance without a proper understanding of the probabilistic nature of these games, and a sensible strategy was to look at what other gambling houses were doing. Gambling house managers would have paid special attention to two aspects: how the games were designed (including odds) by rival peers and the financial success of other gambling businesses. Selective imitation and retention could then shape the games in a “design without a designer” manner, resulting in odds in games of chance that seemed carefully crafted to ensure a house advantage that is sufficient but not excessively large. Note that the application of evolutionary thinking to explain sociocultural phenomena is not a novel approach in the social sciences: media, social movements, and organizational behavior have all been subject to explicit “variation-selection” style evolutionary theorizing (Baum & Singh, 1994; Koopmans, 2004).

While the Darwinian metaphor should not be taken too literally, since strictly speaking the cultural evolution of odds in games of chance may not be adaptive in the biological fitness sense, the process could be viewed as payoff-biased transmission (Kendal et al., 2009), with payoff approximated as monetary profits. It is possible that the gambling houses’ decisions were influenced by other transmission biases, such as the conformist bias (Henrich & Boyd, 1998), in which gambling houses would preferentially copy the odds setup used by the majority of other houses; prestige-bias (Henrich & Gil-White, 2001), wherein the odds from more reputable gambling houses would be selectively imitated; or a combination of multiple learning biases (Hong, 2022a). Gambling houses may also have adopted simple heuristics such as “set the payoff multiplier to be a little less than the number of options” in the flower lottery, and it is difficult to know with certainty from the available historical record. It is also possible that certain individuals may have had a rudimentary sense of quantified uncertainty. However, such knowledge was most likely not articulated, formalized, or systematically transmitted, and therefore did not significantly impact the cultural evolution of odds in games of chance. This is because while odds and profitability were visible, probabilistic knowledge was not, and if a smart manager recognized the probabilistic principles underpinning winning chances and odds, they would likely keep this insight confidential in a competitive market environment.

Probabilistic thinking does not come naturally to humans. The inclination to view uncertain outcomes as inherently random typically requires considerable cultural input, which is unlikely to occur in societies lacking either the knowledge itself, the social structure that facilitates the dissemination of such knowledge, or both. However, this is not to deny humans’ basic understanding of uncertainty. Research in developmental psychology has shown that children as young as 12 months old can perceive and compare the magnitude of uncertainty and are aware that certain events are more likely to occur than others (Téglás & Bonatti, 2016), and people in small-scale, traditional societies clearly understood that divination and magic do not work 100% of the time (Hong, 2022c). Even nonhuman animals have been shown to be sensitive to cues of uncertainty and act accordingly. For example, honeybees and dolphins would selectively opt out of trials in which obtaining a reward is probabilistically unlikely (Perry & Barron, 2013; Smith et al., 1995).^22^ Experienced gamblers certainly have the intuition that achieving a total of 6 with two dice throws is more likely than a total of 2, and that a winning chance of 1 in 30 is more favorable than 1 in 36. However, the critical step of “taming” chance into numerical, calculable entities (Hacking, 1990) presented a significant barrier to the conscious *computation* of expected returns, and thus has important implications for the cultural evolution of the design of games of chance.

While the focus of this paper is on the Chinese case, the cognitive and cultural evolutionary arguments presented may be generally applicable to pre-probabilism societies. Indeed, neither the lack of probabilistic thinking nor the cultural evolutionary dynamics in gambling markets were unique to late-nineteenth/early-twentieth-century China. As long as gamblers do not perceive the outcomes of games of chance as random, and gambling houses are driven by the need to make a profit, odds may appear “designed” due to our evolved psychological tendency for social learning.

We may occasionally overlook the stark contrast between the world we live in today and that of our not-so-distant ancestors, not only in terms of material technologies but also in how we perceive and understand reality. It is ironic that our attitude toward uncertainty has evolved from inarticulate intuitions to an ideology in which quantification and metrics have dominated nearly every aspect of our lives (Muller, 2018). Today, nearly all cultural activities involving significant uncertainties and risks, such as gambling, insurance, annuities, and financial investments, have been subject to meticulous quantification and probabilistic analysis. An appreciation of how our perception of uncertainty has changed can help us better understand human decision-making and how cultural practices may be influenced by individuals’ beliefs and actions in different contexts. Future work may further explore the psychological, social, and cultural factors contributing to folk understanding of chance and uncertainty, and it should apply additional analysis (e.g., cross-cultural comparisons) to check the validity and generalizability of the proposed cultural evolutionary mechanisms on the design of odds in games of chance.

## Data Availability

All data and code used for analysis can be found in https://github.com/kevintoy/games_of_chance\.

## Footnotes

1 Also referred to as “house edge,” a measure of how much the house expects to win in the long term, typically expressed as a percentage of the player’s wager.

2 This is not to suggest that no one in premodern China had any understanding of probability theory. Indeed, the presence of European scholars and missionaries in East Asia during that period makes it plausible that writings on probability theory by European mathematicians could have circulated in the region. However, there are reasons to believe that probabilistic knowledge did not significantly impact the design of cultural artifacts and institutions. Were it otherwise, there would likely be historical records given China’s extensive literary tradition (see the Discussion section for more details).

3 Importantly, deterministic thinking may provide adaptive benefits in certain circumstances (Moore, 1957).

4 I am keenly aware that contrasting the traditional vs. the modern in this way risks positing a false dichotomy. Surely, there are plenty of gamblers in contemporary, modern societies who are as superstitious as their “premodern” counterparts. What I wish to emphasize here is the relative difference in the overall zeitgeist in people’s epistemic orientations (Hong & Henrich, 2021).

5 In reality, gambling houses would intentionally involve human decision-making in some games that could be purely chance-based, as will be discussed in more detail below.

6 In the Chinese context, such fees charged by the house are typically referred to as *choutou* (抽頭).

7 “Surname guessing” is a type of lottery where players bet on the surnames of those who would pass the civil examinations in imperial China (Li, 2015). It is not a game of chance because the players could obtain information about the candidates regarding their likelihood of passing the exam and thus increase their chances of winning.

8 Interested readers may check Elvin (2010), Kidd (2020), and Sambursky (1956) for additional references.

9 Of course, this is not to say that gamblers in modern societies are completely rid of such habits of promiscuous causal thinking; it simply highlights the fact that a supernatural understanding of the causal structure of the world was the dominant mode of thinking in contrast to its marginalized status in most contemporary, modern societies.

10 The gamblers could also attribute divination failures to a lack of sincerity; see Hong et al. (2024).

11 For examples, see Duzhetongxun 讀者通訊 by Wang Junwei 王君偉, in Dameiwanbao 大美晚報, 1933-01-25.

12 These accounts were predominantly written by critics of gambling and should be taken with a grain of salt.

13 See Liankailiuciyuanguishiyelaopodiaojing 連開六次元貴師爺老婆吊頸 by Landudawang 爛賭大王, in Dazhongbao (Macau) 大眾報(澳門), 1943-07-15.

14 See Shidujiupian 十賭九騙, in Nanhuaribao 南華日報, 1937-06-08, and Jingchangdangju genjuezihuadu 員警當局根絕字花毒 in Nanhuaribao 南華日報, 1940-09-10.

15 The categories were determined in consultation with professional historians of China. Obviously, one could come up with alternative classification schemes; the key category here is “house advantage” because it reveals the critics’ understanding of the probabilistic nature of the flower lottery.

16 It is possible that some neighboring non-Chinese gambling houses had utilized probability theory in designing their games, but the linguistic and cultural barriers may have prevented the spread of formal, mathematical knowledge involved in the design of gambling games.

17 Culin’s (1889) description: “A player throws eight dice. If at least three fall like the number bet on, the game keeper pays him eight times, or if six or more are like the number bet on, sixteen times the amount of his stakes. In any other event, the player loses.”

18 For lottery games with “fair” odds, it is likely that a commission fee on winning was charged which was missed in written records.

19 Of course, social learning happens in most market settings, and here I wish to highlight the point that the two opposite decision-making dynamics will nonetheless produce similar outcomes.

20 In modern societies, the state often has a monopoly in operating lotteries and other gambling activities and the house advantage is typically higher (50%) than in a fully competitive gaming markets (Clotfelter & Cook, 1990).

21 Stigler (2022) has shown that after France adopted the Genoese lottery to help government finance, other European nations quickly followed suit and were clearly copying the odds from each other, despite the fact that the probability calculus involved was well-understood by their designers.

22 The underlying cognitive mechanisms via which prelinguistic children and nonhuman animals perceive uncertainty in outcomes of interest is still under scholarly debate. For a recent review, see Leahy & Carey, 2020.
